# Massive hollow catheter thrombus in venovenous extracorporeal membrane oxygenation assisted lung transplantation: A case report

**DOI:** 10.1097/MD.0000000000024235

**Published:** 2021-01-08

**Authors:** Ting Chen, Li Yao, Xiaoqin Fan, Chunyan Zhu

**Affiliations:** a Intensive Care Unit, The Second People's Hospital of Hefei; b Intensive Care Unit, The First Affiliated Hospital of USTC, University of Science and Technology of China, Anhui, China.

**Keywords:** lung transplantation, massive hollow thrombus, venovenous- extracorporeal membrane oxygenation

## Abstract

**Rationale:**

Catheter-related thrombosis is a serious complication of lung transplantation under venovenous extracorporeal membrane oxygenation (ECMO). Although ECMO-related thrombosis is not uncommon, there are few reports of giant hollow catheter thrombosis in lung transplantation under venovenous ECMO (ECMO). Blood loss and transfusion of coagulation factors may promote ECMO-related thrombosis. Hollow catheter thrombus was not detected on ultrasonography performed after initiation of ECMO. Therefore, it is essential to identify, manage, and reduce or avoid such thrombosis.

**Patient concerns:**

We report a rare case of a 43-year-old man with advanced silicosis who developed a massive hollow catheter thrombus during lung transplantation. Anticoagulant therapy did not affect the size of the thrombus.

**Diagnosis:**

Giant hollow catheter thrombosis was diagnosed by ultrasonography. Thrombosis from the right external iliac vein to the inferior vena cava was found in the shape of the ECMO pipe.

**Interventions:**

Heparin was prescribed as an anticoagulant.

**Outcomes:**

Anticoagulant therapy did not affect the size of the thrombus during 2 weeks. The patient developed an infection and died of multiple organ failure.

**Conclusion:**

It is uncommon for massive hollow thrombus to occur during venovenous-ECMO-assisted lung transplantation. Fibrinogen and prothrombin complexes promote the formation of thrombus, and the measurement of the wall thickness of ECMO catheter may help to detect such thrombus.

## 1 Introduction

Extracorporeal membrane oxygenation (ECMO) saves time when treating reversible heart and lung diseases, and has been used in a wide range of conditions since the landmark CESAR trial.^[1]^ However, ECMO frequently causes vascular complications such as hemorrhage and thrombosis, which may result in serious sequelae and even death.^[2]^ The causes of ECMO-related thrombosis may be puncture injury, old age, and ECMO duration. ECMO-related thrombosis reportedly occurs in 85.4% of cases,^[3]^ but there have been no reports of massive hollow catheter thrombosis during venovenous ECMO (VV-ECMO)-assisted lung transplantation.

## 2 Case report

**Figure 1:**
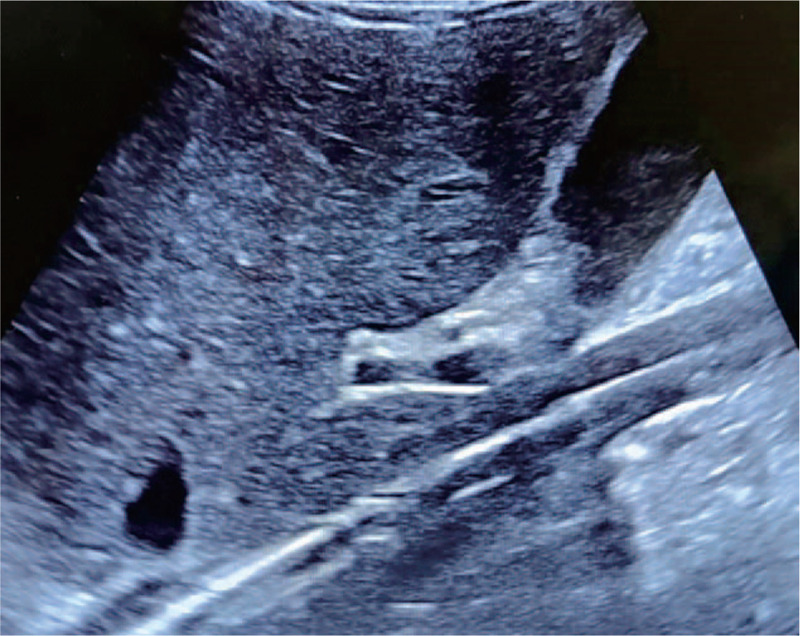
Inferior vena cava catheter shadow before extracorporeal membrane oxygenation weaning.

**Figure 2:**
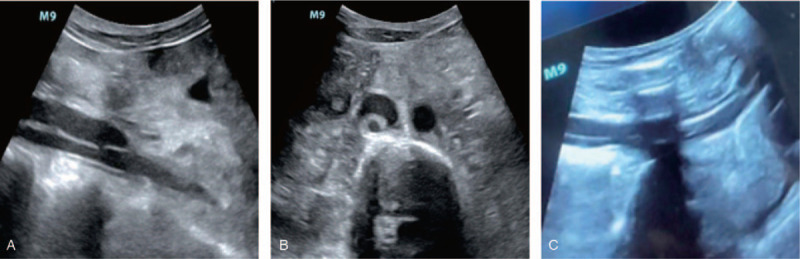
Inferior vena cava thrombosis after extracorporeal membrane oxygenation removal (A: vertical axis; B: cross axle); external iliac vein thrombosis after ECMO withdrawa (C).

A 43-year-old man (height 172 cm, weight 42 kg) underwent left lung transplantation under VV-ECMO. He had no history of hypertension or diabetes. The platelet count, coagulation tests, and liver and kidney function tests were all within normal ranges. The ECMO catheter (Duraflo; Edwards Lifesciences, Irvine, CA) was inserted via the right internal jugular vein. A 17-f catheter was selected for the right internal jugular vein at a depth of adjustment between the superior vena cava and right atrium, and a 19-f catheter was selected for the right femoral vein at a depth of adjustment between the superior vena cava and right atrium. The initial ECMO rotation speed was 3500 r/min and the flow rate was 4.2 L/min. A large amount of blood oozed from the wound surface, and 10,500 mL of blood was lost when the left lung pleura was stripped. The ACT was 162 second. The ECMO tube was shaken, the ECMO rotation speed was decreased to 2000 r/min, and the flow rate was decreased to 1.0 to 1.2 l/min for the remaining 1.5 hours of surgery. Intraoperatively, the patient was administered 5500 mL of autologous blood, 20 U of red blood cells, 2300 mL of plasma, 10.5 U of cryoprecipitate, and 1 U of platelets. In addition, 3 g of human fibrinogen (Green Cross Biological Products Co., Ltd.) and 800 U of prothrombin complex (Hualan Bioengineering Co., Ltd. China) were infused within 32 minutes. Postoperatively, the ECMO speed was 3000 r/min and the flow rate was 3.6 L/min. In accordance with ECMO anticoagulant management procedures, heparin was administered to maintain an APTT of 45 to 60 second. If the hemoglobin was less than 8.0 g/L, red blood cell suspension was infused. If the platelet count was less than 30 × 10^9^/L, platelets were infused. The drainage volume of the closed thoracic drainage tube decreased from 1000 mL on postoperative day 1 to 240 mL on postoperative day 2 and 80 mL on postoperative day 3. After the ECMO had been stopped for 2 hours, the heart rate, blood pressure, and peripheral oxygen saturation remained unchanged; blood gas analysis showed a pH of 7.38, PO_2_ of 103 mm Hg, and PCO_2_ of 40.2 mm Hg. Bedside ultrasonography performed before ECMO weaning did not reveal any ECMO lumen abnormality (Fig. 1). Thus, the patient was removed from the VV-ECMO machine. Six hours after ECMO withdrawal, thrombosis from the right external iliac vein to the inferior vena cava was found in the shape of the ECMO pipe (Fig. 2). Heparin was prescribed as an anticoagulant. Two weeks later, the patient developed a severe infection and multiple organ failure. The patient died 1 week after discharge.

## 3 Discussion

A 2009 study showed that ECMO significantly reduces the H1N1 mortality rate,^[1]^ and ECMO has since been widely used in the rescue of critically ill patients. However, ECMO often results in complications, especially when used for long periods. Common vascular complications of ECMO include thrombus and hemorrhage, which are the most frequent cause of disability and death.^[2]^ ECMO may cause large amounts of blood to ooze from the wound surface due to the activation of coagulation and consumption of coagulation factors. However, reductions in the ECMO speed and the infusion of coagulation factors may promote thrombosis. Thus, ECMO-related vascular complications are related to anticoagulant management, surgery, and the infusion of blood products.^[4]^

Major surgery activates both the coagulation and fibrinolysis systems, similarly to uncontrolled disseminated intravascular coagulation. The coagulation system is activated by exogenous blood components and the surface of the ECMO tube or thoracic surgery. The fibrinolytic system is activated by the release of tissue plasminogen activator, which can be promoted by ECMO tube-mediated contact activation of fibrinogen and thrombin. In the present case, we administered prothrombin complex to promote thrombin formation. Previous in vitro studies^[5]^ have showed that prothrombin complex promotes thrombin formation without the need for enolase on the platelet surfaces. Thrombin further accelerates the formation of fibrinogen, fibrin, and thrombi. The inner surface of the ECMO pipeline is coated with heparin, and the anticoagulant activity of heparin increases 1000-fold after combining with antithrombin III. Thus, thrombi do not easily form on the inner surface of the ECMO pipeline. However, the outer surface is not coated with heparin, which makes it easier for thrombosis to occur. In addition, due to the large amount of bleeding and ECMO tube shaking in the present case, the ECMO speed and the flow rate were decreased intraoperatively, which increased the contact time between the blood components and ECMO tube wall, and increased the likelihood of the ECMO tube wall adhering to the vascular wall. Therefore, we believe that the formation of the massive hollow thrombus was closely related to the infusion of fibrinogen and prothrombin complex; blood loss, low ECMO speed, and anticoagulation without heparin may also have played important roles. A previous study also reported that prothrombin complex promotes thrombosis.^[6]^

An ECMO-related thrombus is difficult to detect on ultrasonography, as it is easily mistaken for the ECMO tube wall. In the present case, 2-dimensional ultrasonography showed an echo of the vessel wall, intima, and lumen, while color doppler showed blood flow through the thrombus. As the thrombosis from the inferior vena cava to the right external iliac vein did not block the blood flow, and there was no inferior vena cava obstructive syndrome. The common femoral vein was supported by the ECMO tube, so that the venous lumen could not be compressed; the vein was completely covered by the ECMO tube, and so was mistaken for the wall of the ECMO tube. At present, there is no good way to identify such thrombi using ultrasonography. ECMO-related thrombosis may be judged by repeated measurements of the thickness of the ECMO tube wall, but this requires further clinical practice. Griffith et al^[7]^ reported the successful removal of an ECMO-related thrombus using the AngioVac system. In the present case, the selected treatment was anticoagulant therapy. However, the thrombus size did not decrease before the patient was discharged.

In conclusion, we reported a rare case of massive hollow ECMO-related thrombosis. The present case highlights the need for caution when administering fibrinogen and prothrombin complex in ECMO. Furthermore, thrombosis may be avoided by coating the outer surface of the ECMO tube with heparin. Monitoring the change in ECMO tube thickness on ultrasonography may help in the early detection of thrombus formation. However, **hollow catheter thrombus was not detected on ultrasonography when ECMO is running**. It is vital to identify, manage, and reduce or avoid hollow ECMO related thrombosis as early as possible.

## Acknowledgments

We thank Kelly Zammit, BVSc, from Liwen Bianji, Edanz Editing China (www.liwenbianji.cn/ac), for editing the English text of a draft of this manuscript.

Written informed consent was obtained from the patient's guardian to publish the details of the case.

## Author contributions

Ting Chen and Chunyan Zhu designed the paper and wrote the first draft. All authors participated in drafting and reviewing the manuscript. All authors read and approved the final manuscript.

**Conceptualization:** Ting Chen.

**Data curation:** Ting Chen, Xiaoqin Fan.

**Formal analysis:** Ting Chen.

**Investigation:** Ting Chen, Chunyan Zhu.

**Methodology:** Ting Chen, Li Yao.

**Project administration:** Ting Chen, Chunyan Zhu.

**Resources:** chunyan zhu.

**Supervision:** Li Yao, Chunyan Zhu.

**Validation:** Xiaoqin Fan.

**Writing – original draft:** Ting Chen.

**Writing – review & editing:** Ting Chen, Chunyan Zhu.
